# The Effect of Metformin on Plasma Prolactin Levels in Young Women with Autoimmune Thyroiditis

**DOI:** 10.3390/jcm12113769

**Published:** 2023-05-31

**Authors:** Robert Krysiak, Karolina Kowalcze, Andrzej Madej, Bogusław Okopień

**Affiliations:** 1 Department of Internal Medicine and Clinical Pharmacology, Medical University of Silesia, 40-752 Katowice, Poland; bokopien@sum.edu.pl; 2 Department of Pediatrics in Bytom, School of Health Sciences in Katowice, Medical University of Silesia, Stefana Batorego 15, 41-902 Bytom, Poland; kkowalcze@sum.edu.pl; 3 Department of Pharmacology, Faculty of Medicine, University of Technology, Academy of Silesia, Park Hutniczy 3-5, 41-800 Zabrze, Poland; andrzejmadej@o2.pl

**Keywords:** autoimmune thyroiditis, hyperprolactinemia, insulin resistance, lactotrope secretory function

## Abstract

Metformin decreases elevated prolactin levels, which are frequently found in patients with thyroid disorders. The aim of this study was to investigate whether thyroid autoimmunity modulates the impact of metformin on lactotrope secretory function. This study compared two matched groups of young women with prediabetes and mild-to-moderate prolactin excess: 28 subjects with coexisting euthyroid autoimmune thyroiditis (group 1) and 28 individuals without thyroid disorders (group 2), treated for six months with metformin (3 g daily). Thyroid antibody titers, glucose homeostasis markers, prolactin, thyrotropin, free thyroid hormones, FSH, LH, ACTH, IGF-1 and hsCRP were assessed at the beginning and at the end of the study. At entry, the study groups differed in antibody titers and hsCRP levels. Although the improvement in glucose homeostasis and the decrease in hsCRP levels were observed in both study groups, they were more pronounced in group 2. Only in group 2 did metformin reduce circulating prolactin levels (both total and monomeric). Prolactin-lowering properties of metformin positively correlated with baseline prolactin levels, baseline antibody titers (in group 1) and with the degree of reduction in hsCRP levels. The obtained results suggest that autoimmune thyroiditis may attenuate the impact of metformin on lactotrope secretory function.

## 1. Introduction

Recent studies have shown that metformin, the most commonly prescribed antidiabetic drug worldwide [1], inhibits the secretory function of overactive lactotropes [2,3,4,5,6,7]. The drug decreased circulating prolactin levels independently of the reason for prolactin excess: in microprolactinomas [2], iatrogenic hyperprolactinemia [3,4,5], empty sella syndrome [2], traumatic brain injury [2] and polycystic ovary syndrome [6], and in all these conditions the degree of reduction in prolactin levels correlated with baseline hormone levels. The drug was also found to reduce prolactin levels in individuals treated with moderate doses of bromocriptine [2,7] but not in subjects with prolactin-secreting tumors resistant to high-dose cabergoline treatment [8]. The prolactin-lowering effect of metformin was more pronounced in women than men [9], depended on estrogen levels [10], and was stronger in subjects receiving high-dose than moderate-dose treatment with this agent [4]. The reduction in prolactin levels seems to be limited to a decrease in monomeric prolactin because the drug exerted a neutral effect on a high-molecular-mass non-bioactive isoform of this hormone (macroprolactin) [11]. Prolactin-lowering properties are probably a consequence of the fact that the pituitary gland is located outside the blood–brain barrier, and anterior pituitary cells are exposed to metformin present in the peripheral blood and deposited in this brain region [12].

Autoimmune thyroiditis, also referred to as Hashimoto’s thyroiditis, is the most prevalent organ-specific autoimmune disorder worldwide and the leading cause of hypothyroidism in iodine-replete areas [13,14]. It is assumed that prolactin interferes with immune system modulation, mainly inhibiting the negative selection of autoreactive B lymphocytes and stimulating the maturation of T cells [15,16]. Increased prolactin production may contribute to the development and perpetuation of several autoimmune diseases, including autoimmune thyroid disease [15]. Thyroid antibodies are more prevalent in subjects with untreated prolactin excess than in patients with prolactinoma treated with cabergoline [17]. Moreover, up to 40% of patients with overt primary hypothyroidism and up to 22% of patients with subclinical hypothyroidism can present with elevation of prolactin levels [18]. Moreover, both hyperprolactinemia and autoimmune thyroiditis occur several times more frequently in women than men and are highly prevalent in young women [13,18].

Only one previous study indirectly assessed the impact of autoimmune thyroiditis on the pituitary effects of metformin. Metformin-induced reduction in thyrotropin levels was observed in women with subclinical hypothyroidism of both autoimmune and non-autoimmune origin coexisting with polycystic ovary syndrome and did not depend on titers of thyroid antibodies [19]. However, recently, the same research group has reported that women with Hashimoto’s thyroiditis were partially resistant to the cardiometabolic effects of atorvastatin [20]. Therefore, the current study was aimed at investigating whether thyroid autoimmunity modulates the impact of metformin on circulating levels of prolactin, other anterior pituitary hormones and their downstream hormones in young women with prolactin excess.

## 2. Materials and Methods

The study was performed in accordance with the 1964 Helsinki Declaration, and the study protocol was approved by the local review board. All patients gave written informed consent after receiving a comprehensive explanation of the nature of the study.

### 2.1. Study Population

The study population was recruited among women of reproductive age (20–50 years old) with mildly or moderately elevated plasma prolactin levels (between 30 and 70 ng/mL) and prediabetes (fasting glucose between 100 and 125 mg/dL and/or 2 h post-glucose load between 140 and 199 mg/dL), despite complying for at least three months with the lifestyle modification. Potential participants of this prospective matched cohort study were initially supervised by local healthcare providers cooperating with our research team and were referred to our clinical, which is a tertiary referral center for patients with metabolic and hormonal disorders. Only euthyroid women (thyrotropin between 0.4 and 4.5 mIU/L, free thyroxine between 10.2 and 21.3 pmol/L and free triiodothyronine between 2.2 and 6.4 pmol/L) were included. The participants were divided into two groups: group 1 included individuals with euthyroid autoimmune thyroiditis, defined as thyroid peroxidase antibody (TPOAb) titers above 100 U/mL and the presence of sonographic features of autoimmune thyroiditis; and group 2 consisted of women without thyroid pathology. The number of individuals in each group (n = 28) exceeded the minimum sample size. Assuming a significance level of 0.05 and a power of 80%, at least 25 patients per group must have been included to detect a 20% between-group difference in plasma levels of total prolactin (the primary endpoint). These patients were selected from larger groups of potential participants (50 with autoimmune thyroiditis and 74 without thyroid pathology) on the basis of a computer algorithm, aimed at obtaining two populations matched for age, body mass index, glucose levels, insulin sensitivity and prolactin levels. In order to limit the impact of seasonal fluctuations in the outcome variables, 27 women (14 in group 1 and 13 in group 2) were recruited between February and March, while the remaining ones in August or September.

Because individuals with tumor-induced hyperprolactinemia require an individual approach, we excluded women with prolactin-secreting tumors, mixed pituitary tumors (secreting prolactin and other anterior pituitary hormones) and pseudoprolactinoma. We also excluded subjects meeting at least one of the following criteria: positive serum antibodies against thyrotropin receptor, macroprolactinemia, polycystic ovary syndrome, diabetes, other endocrine disorders, cardiovascular disease, kidney insufficiency, liver failure, anemia, oncological diseases, malabsorption syndromes, other inflammatory or autoimmune diseases, any other serious disorders, pregnancy or lactation, a desire to become pregnant, hormonal contraception, concomitant treatment with any drugs (except for antipsychotics) and poor patient compliance.

### 2.2. Study Design

Over the entire study period (six months), all participants were treated with metformin. In the first week, they received 500 mg of this drug twice a day, during or immediately after meals. For the following two weeks, the metformin dose was increased to 1 g twice a day. From week 4 onward, the daily dose of metformin was 3 g and was administered in three equal doses. The patients were also requested to follow the lifestyle modification (total fat intake <30% of total energy intake, saturated fat intake <7% of energy consumed, cholesterol intake <200 mg per day, ≥15 g per 1000 kcal, moderate to vigorous exercise for at least 30 min per day). Any antipsychotics that the participants were taking before enrollment in the study remained at the same dosage over the entire study period. Hormonal drugs (including contraceptives), immunosuppressive agents (including systemic glucocorticoids) and drugs known to affect glucose homeostasis or anterior pituitary function were not allowed during the study. Short-term (for less than seven days) use of other agents was accepted only if such treatment was terminated at least four weeks before the end of the study. The withdrawal criteria were as follows: serious adverse effects, consent withdrawal, changes in pharmacological treatment (other than mentioned above) and pregnancy. In agreement with Food and Drug Administration recommendations [21], serious adverse effects were defined as a life-threatening adverse event, inpatient hospitalization or prolongation of existing hospitalization (for >24 h), a persistent or significant incapacity or substantial disruption of the ability to conduct normal life functions, an event jeopardizing the subject or an event requiring medical or surgical intervention to prevent one of these outcomes. Compliance was measured at each visit taking place every eight weeks by counting the remaining pills and analyzing eating diaries and diaries in which the patients recorded all their activities.

### 2.3. Laboratory Assays

All measurements were carried out in duplicate on the first and last day of the study. Venous blood samples were obtained from the antecubital vein between 8.00 and 9.00 a.m. after an overnight 12 h fasting in the early follicular phase (days 2 and 5, counting from the first day of the last menstrual period). Titers of TPOAb and thyroglobulin antibodies (TgAb), as well as plasma levels of prolactin, insulin, thyrotropin, free thyroid hormones (free thyroxine and free triiodothyronine) and gonadotropins (follicle-stimulating hormone (FSH) and luteinizing hormone (LH)) were measured using acridinium ester technology (ADVIA Centaur XP Immunoassay System, Siemens Healthcare Diagnostics, Munich, Germany). Prior to venipuncture, all participants had been resting for at least 30 min in the seated position. All measurements were performed in duplicate to ensure the consistency of assessments. Prolactin concentrations were measured both before (total prolactin) and after (monomeric prolactin) polyethylene glycol precipitation as previously described [22]. To correct for the dilution with polyethylene glycol, the post-polyethylene glycol prolactin concentration was determined by multiplying the prolactin result by 2. Macroprolactin content was calculated by subtracting monomeric prolactin from total prolactin. Plasma glucose was measured using standard enzymatic methods (Roche Diagnostics, Basel, Switzerland). Concentrations of adrenocorticotropic hormone (ACTH), insulin-like growth factor-1 (IGF-1) and high-sensitivity C-reactive protein (hsCRP) were measured by solid-phase enzyme-labelled chemiluminescent immunometric assays (Immulite, Siemens, Munich, Germany). The homeostatic model assessment 1 of insulin resistance (HOMA1-IR) was calculated as follows: fasting glucose (mg/dL) × fasting insulin (mU/L)/405.

### 2.4. Statistical Analysis

All data were log-transformed to improve the approximation to a normal distribution. Between-group comparisons at the same time point and comparisons of percentage changes from baseline were performed using Student’s *t* tests for independent samples, while within-group comparisons were performed using Student’s paired *t* test. Categorical variables were analyzed with the χ^2^ test. Correlations were assessed using Pearson’s correlation coefficient (r). The data were analyzed with a predetermined level of significance set to a *p*-value corrected for multiple testing below 0.05.

## 3. Results

At entry, the study groups differed in TPOAb titers, TgAb titers and hsCRP levels. There were no between-group differences in age, smoking habits, reasons for hyperprolactinemia, the body mass index, blood pressure, glucose, HOMA1-IR, thyrotropin, free thyroid hormones, total prolactin, monomeric prolactin, macroprolactin, FSH, LH, ACTH and IGF-1 (Table 1 and Table 2).

During the follow-up period, no patient developed serious or unexpected adverse events. Two women from group 1 complained of a metallic taste in their mouth and nausea. In turn, two women from group 2 experienced a loss of appetite and increased flatulence. However, these adverse effects were mild and transient (disappeared within three weeks), and all patients completed the study. All participants complied with treatment recommendations and adhered to the recommendations on diet and physical activity.

**Figure 1:**
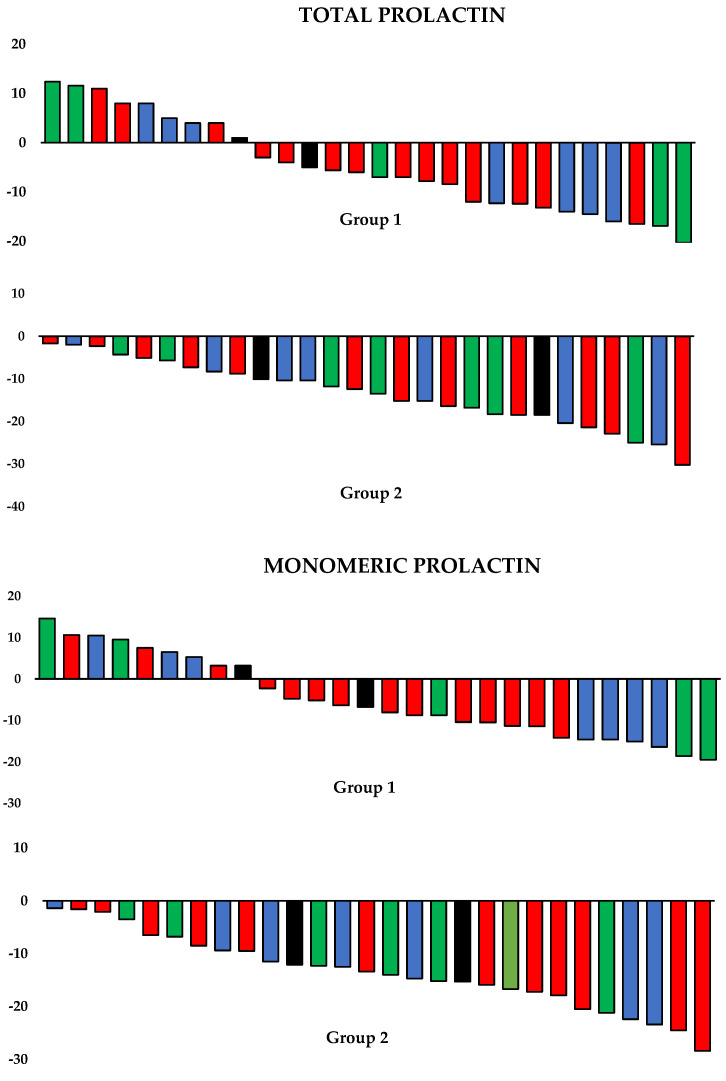
**Percentage changes from baseline in prolactin levels.** Group 1: hyperprolactinemic women with autoimmune thyroiditis; Group 2: hyperprolactinemic women without thyroid pathology. Percentage changes from baseline were calculated by subtracting the baseline value from the follow-up value and dividing the result by the baseline value and multiplying by 100. Reasons for hyperprolactinemia: antipsychotic-induced (red color), empty sella syndrome (green color), traumatic brain injury (blue color) and idiopathic (black color).

Metformin reduced plasma glucose, HOMA1-IR and hsCRP levels in both groups of patients. Only in group 2 did metformin reduce circulating levels of total and monomeric prolactin. There were no significant differences between baseline and follow-up body mass index, thyroid antibody titers and levels of thyrotropin, free thyroid hormones, macroprolactin, FSH, LH, ACTH and IGF-1. Both groups of patients differed in follow-up values of glucose, HOMA1-IR, antibody titers, total prolactin, monomeric prolactin and hsCRP. Percentage changes from baseline in glucose, HOMA1-IR, total prolactin, monomeric prolactin, FSH, LH and hsCRP were more pronounced in group 2 than in group 1 (Table 2). The decrease in total and monomeric prolactin levels was observed in all 28 patients belonging to group 2, independently of the reason for hyperprolactinemia (Figure 1).

At baseline, thyroid antibody titers in group 1 positively correlated with hsCRP levels (TPOAb: r = 0.48, *p* < 0.0001; TgAb: r = 0.43, *p* = 0.0004). The impact of metformin on plasma prolactin positively correlated with baseline prolactin levels (group 1—total prolactin: r = 0.38, *p* = 0.0023, monomeric prolactin: r = 0.34, *p* = 0.0255; group 2—total prolactin: r = 0.42, *p* = 0.0004, monomeric prolactin: r = 0.47, *p* = 0.0001) and with the degree of reduction in hsCRP levels (group 1—total prolactin: r = 0.38, *p* = 0.0014, monomeric prolactin: r = 0.40, *p* = −0.0008; group 2—total prolactin: r = 0.32, *p* = 0.0321, monomeric prolactin: r = 0.37, *p* = 0.0078). In group 1, there were inverse correlations between the impact on prolactin levels and baseline antibody titers (TPOAb—total prolactin: r = −0.41, *p* = 0.0006, monomeric prolactin: r = −0.48, *p* = 0.0001; TgAb—total prolactin: r = −0.30, *p* = 0.0412, monomeric prolactin: r = −0.32, *p* = 0.0382). The impact of metformin on HOMA1-IR positively correlated with the changes in hsCRP (group 1—r = 0.34, *p* = 0.0187; group 2—r = 0.38, *p* = 0.0022). In group 2, there were positive correlations between the reduction in prolactin levels and the changes in FSH (total prolactin: r = 0.29, *p* = 0.0425, monomeric prolactin: r = 0.32, *p* = 0.0362) and in LH (total prolactin: r = 0.35, *p* = 0.0121, monomeric prolactin: r = 0.37, *p* = 0.0085). Lastly, metformin-induced reduction in HOMA1-IR positively correlated with the degree of reduction in total (group 1—r = 0.30, *p* = 0.0427; group 2—r = 0.28, *p* = 0.0498) and monomeric prolactin (group 1—r = 0.31, *p* = 0.0398; group 2—r = 0.34, *p* = 0.0241).

## 4. Discussion

The current study has shown that six-month metformin treatment decreased prolactin levels in hyperprolactinemic women without thyroid pathology and that the strength of this effect depended on the degree of prolactin excess. The decrease in prolactin levels resulted from the reduction in monomeric prolactin and was not associated with any changes in macroprolactin concentrations. Thus, metformin treatment should be taken into consideration in patients with hyperprolactinemia secondary to antipsychotic use, empty sella syndrome, traumatic brain injury or of unknown etiology in case of resistance, poor tolerance or contraindications to dopaminergic agents. Prolactin-lowering properties of metformin seem particularly important in case of iatrogenic prolactin excess, which can be very difficult to treat. Antipsychotics often cause symptomatic hyperprolactinemia resulting from markedly elevated prolactin levels, while the addition of dopaminergic agents can aggravate psychosis, increase hallucinations and aggressiveness or cause the appearance of abnormal involuntary movements in patients treated with antipsychotics [23]. Future research is required to establish whether metformin treatment is also beneficial for patients with prolactinoma, who, for ethical reasons, did not participate in the study (organic causes of prolactin excess always require specific treatment: dopaminergic agents or surgery) [24].

Dose-dependent gastrointestinal intolerance has been reported in about one-fourth of patients receiving this agent [25]. In the current study, metformin administered at the daily dose of 3 g was well tolerated and not associated with any serious adverse events. Only four patients (14%) developed mild and transient gastrointestinal side effects and no patient experienced serious adverse effects. Good tolerance of metformin was most likely related to the young age of the study population, strict exclusion criteria regarding comorbidities and comedications, slow metformin dose titration and a high level of adherence to the study population.

The daily dose of metformin in our study exceeded that used by the participants of the Diabetes Prevention Project (1.7 g daily), a landmark clinical trial demonstrating that metformin reduces progression to diabetes [26]. However, previous studies reported that the impact of metformin on lactotrope secretory function was significant after treatment with 2.55–3 g of this agent but not after treatment with 1.7 g daily, recommended to subjects at high diabetes risk [2,4]. Thus, the inhibitory effect on pituitary secretory function seems to be dose-dependent. Interestingly, the pituitary content of this drug in metformin-treated rats with plasma metformin concentrations the same as in the plasma of subjects receiving high-dose metformin treatment was higher than in other rat brain regions (the hypothalamus, frontal cortex, hippocampus, cerebellum, striatum and olfactory bulbs) [12,27,28].

Despite no significant differences between baseline and follow-up gonadotropin concentrations, both groups differed in the percentage changes from baseline in FSH and LH levels, more pronounced in women without thyroid disorders. This finding, low-normal baseline levels of FSH and LH and the presence of correlations between the impact of treatment on both gonadotropins and the degree of reduction in total and monomeric prolactin, suggests that slight changes in gonadotropin levels in women without thyroid disorders reflect the partial normalization of hypothalamic–pituitary–gonadal axis activity, suppressed by chronic prolactin excess [29]. However, although the current study included young women not using hormonal contraception and all participants received metformin, the drug inducing ovulation and improving contraception rates in women with another endocrine disease—polycystic ovary syndrome [30], there were no cases of incident pregnancies over the entire study period. Not including women desiring pregnancy undoubtedly contributed to this finding. However, the complete lack of unplanned pregnancies in 56 young women participating in the study may be partially explained also by the fact that the treatment-induced changes in plasma gonadotropins were small and that, because of an only moderate effect on prolactin levels, follow-up levels of this hormone were still above the upper limit of normal.

More importantly, this study has shown for the first time that prolactin-lowering properties of metformin were absent in individuals with coexisting Hashimoto’s thyroiditis. Owing to the selection procedure, both groups did not differ with regard to baseline hormone levels and reasons for prolactin excess. The study groups were also matched for age, body mass index and glucose homeostasis. Finally, the percentage of patients receiving antipsychotic drugs, the only agents used by the participants, was similar in both groups. A neutral effect on prolactin levels was observed despite the fact that thyrotropin and free thyroid hormone levels were within the reference range, indicating that this effect is related to thyroid autoimmunity itself, present in one of the study arms, and cannot be attributed to resultant thyroid hypofunction. Our findings suggest that metformin action on the secretory function of lactotropes may be counterbalanced by systemic inflammation associated with Hashimoto's thyroiditis. Baseline levels of hsCRP, which is a reliable marker of systemic inflammation [31], higher in women with thyroiditis than in women without thyroid pathology, correlated with antibody titers. Moreover, metformin-induced reduction in hsCRP was more pronounced in women without thyroid pathology than in women with thyroiditis and correlated with the impact on both total and monomeric prolactin. Pituitary effects of metformin are postulated to be secondary to its action on adenosine 5′-monophosphate-activated protein kinase, an enzyme playing a central role in regulating energy homeostasis [32,33]. Interestingly, inflammation inhibited the adenosine 5′-monophosphate-activated protein kinase pathway [34], and a similar effect on this pathway was induced by various proinflammatory cytokines [35]. Relatively high follow-up values of hsCRP and markedly elevated follow-up antibody titers indicate that in metformin-treated women with Hashimoto’s thyroiditis the proinflammatory state persists, neutralizing the direct effect of this drug on lactotrope secretory function.

Another interesting observation was between-group differences in metformin action on glucose homeostasis markers, stronger in women without thyroid pathology. Taking into consideration that prolactin impairs glucose tolerance and insulin sensitivity [36], they may be secondary to differences in the impact on prolactin levels. This explanation can be supported by the presence of positive correlations between treatment-induced changes in plasma prolactin and in HOMA1-IR. However, these correlations were not strong, and therefore differences in the impact on glucose homeostasis are probably partially prolactin-independent. They may be also attributed to interactions between metformin and the proinflammatory state at the level of GLUT4, which is the main transporter of glucose in muscle and fat cells and is activated by metformin [37]. In line with this explanation, tumor necrosis factor-α, interleukin-1ß and interferon-γ, secreted in increased amounts by proinflammatory cells (activated monocytes and lymphocytes) of subjects with euthyroid autoimmune thyroiditis [38], were found to inhibit GLUT4 translocation and membrane expression [39,40,41] and may counterbalance the impact of metformin, which is an activator of this transporter [37]. Moreover, the effect of metformin on HOMA1-IR correlated with the reduction in hsCRP.

A neutral effect on thyroid antibody titers is in apparent contradiction with the results of a meta-analysis by Jia et al. [42]. These authors calculated that metformin treatment reduced TPOAb and TgAb titers in individuals with euthyroid Hashimoto’s thyroiditis and subclinical autoimmune thyroiditis. However, their meta-analysis was based almost exclusively on the results of our previous studies, which were similar to those obtained in the current one. Because the antibody-lowering effect of metformin was never the primary endpoint of our studies, they were probably underpowered to detect a significant decrease in TPOAb and TgAb titers. Thus, metformin probably decreases thyroid antibody titers in individuals with Hashimoto’s thyroiditis, but this effect seems to be relatively weak and the question of whether metformin prevents or delays the progression of autoimmune thyroid disease requires further research.

The obtained results allow us to draw additional practical conclusions. Less pronounced changes in plasma glucose levels and HOMA1-IR suggest that metformin may be less effective in the prevention of diabetes in individuals in whom prediabetes coexists with autoimmune thyroiditis than in prediabetic patients without thyroid disorders. Because weak effects of metformin were observed in euthyroid Hashimoto’s thyroiditis, while thyroid hypofunction, independently of its cause, often results in prolactin excess and insulin resistance [18,43], individuals with untreated autoimmune hypothyroidism may be poor candidates for metformin treatment. Lastly, because the impact on total and monomeric prolactin inversely correlated with thyroid antibody titers, the inhibitory effect of metformin on prolactin levels may be partially restored in women concomitantly treated with agents reducing TPOAb and TgAb titers, such as selenium, vitamin D, myoinositol and levothyroxine [44,45].

Some shortcomings of the study should be kept in mind. Owing to the small sample size, our findings should be interpreted as hypothesis-generating rather than definitive conclusions. Because the study population included a heterogenous group of women with prolactin excess, it cannot be completely ruled out that the impact on prolactin levels partially depends on the reason for hyperprolactinemia. The impact of metformin on plasma prolactin may be also different in women with tumor-induced and/or severe hyperprolactinemia, not participating in the current study. Lastly, macroprolactin was measured following polyethylene glycol precipitation, while the gold standard for its detection is gel filtration chromatography [46].

## 5. Conclusions

Metformin treatment decreased total and monomeric prolactin levels only in women without thyroid disorder but not in euthyroid women with Hashimoto’s thyroiditis. Between-group differences in the impact on plasma prolactin were accompanied by different effects of metformin on glucose homeostasis markers, gonadotropins and hsCRP, less pronounced in individuals with thyroiditis. Our findings suggest that autoimmune thyroiditis may attenuate metformin action on lactotrope secretory function and that this effect is associated with thyroid autoimmunity itself, not with thyroid hypofunction. Owing to the preliminary nature of the current study, the obtained results should be verified in a full-scale clinical trial.

## Figures and Tables

**Table 1:** Baseline characteristics of patients.

Variable	Group 1	Group 2	*p*-Value
**Number** (n)	28	28	-
**Age** (years)	35 ± 7	34 ± 8	0.6207
**Smokers** (%)/**number of cigarettes a day** (n)/**duration of smoking** (months)	25/9 ± 4/125 ± 30	29/10 ± 5/128 ± 26	0.6428
**Reasons for prolactin excess** (%)**: drug-induced hyperprolactinemia/empty sella syndrome/traumatic brain injury/idiopathic hyperprolactinemia**	50/18/25/7	43/25/25/7	0.7025
**Body mass index** (kg/m^2^)	24.5 ± 4.0	24.1 ± 4.6	0.7298
**Systolic blood pressure** (mmHg)	128 ± 14	126 ± 12	0.5684
**Diastolic blood pressure** (mmHg)	83 ± 5	82 ± 4	0.4121

Group 1: hyperprolactinemic women with autoimmune thyroiditis; Group 2: hyperprolactinemic women without thyroid pathology. Unless otherwise stated, the data are presented as the mean ± standard deviation.

**Table 2:** The effect of metformin on the investigated variables in the study population.

Variable	Group 1	Group 2	*p*-Value (1 vs. 2)
**Body mass index** (kg/m^2^)			
*Baseline*	24.5 ± 4.0	24.1 ± 4.6	0.7298
*Follow-up*	23.8 ± 3.9	23.0 ± 4.2	0.4634
*p-value (follow-up* vs. *baseline)*	0.5102	0.3542	-
*Percentage changes from baseline*	−3 ± 5	−5 ± 6	0.1811
**Glucose** (mg/dL)			
*Baseline*	109 ± 12	108 ± 11	0.7464
*Follow-up*	103 ± 10	98 ± 8	**0.0436**
*p-value (follow-up* vs. *baseline)*	**0.0470**	**0.0003**	-
*Percentage changes from baseline*	−6 ± 3	−9 ± 4	**0.0025**
**HOMA1-IR** (%)			
*Baseline*	3.9 ± 1.0	3.8 ± 1.2	0.7361
*Follow-up*	3.1 ± 0.8	2.2 ± 0.6	**<0.0001**
*p-value (follow-up* vs. *baseline)*	**0.0017**	**<0.0001**	**-**
*Percentage changes from baseline*	−21 ± 15	−42 ± 20	**<0.0001**
**TPOAb** (U/mL)			
*Baseline*	902 ± 268	16 ± 9	**<0.0001**
*Follow-up*	788 ± 290	15 ± 8	**<0.0001**
*p-value (follow-up* vs. *baseline)*	0.1324	0.6621	-
*Percentage changes from baseline*	−13 ± 10	−7 ± 30	0.3199
**TgAb** (U/mL)			
*Baseline*	860 ± 310	17 ± 8	**<0.0001**
*Follow-up*	750 ± 282	16 ± 8	**<0.0001**
*p-value (follow-up* vs. *baseline)*	0.1706	0.6419	-
*Percentage changes from baseline*	−13 ± 12	−6 ± 28	0.2293
**Thyrotropin** (mU/L)			
*Baseline*	2.6 ± 0.9	2.5 ± 1.0	0.6956
*Follow-up*	2.4 ± 0.7	2.2 ± 0.8	0.3240
*p-value (follow-up* vs. *baseline)*	0.3574	0.2206	-
*Percentage changes from baseline*	−8 ± 8	−12 ± 11	0.1255
**Free thyroxine** (pmol/L)			
*Baseline*	14.9 ± 2.5	15.5 ± 2.8	0.4015
*Follow-up*	15.7 ± 3.0	16.2 ± 3.2	0.5489
*p-value (follow-up* vs. *baseline)*	0.2832	0.3875	-
*Percentage changes from baseline*	5 ± 8	5 ± 10	1.0000
**Free triiodothyronine** (pmol/L)			
*Baseline*	3.5 ± 0.7	3.5 ± 0.8	1.0000
*Follow-up*	3.6 ± 0.7	3.7 ± 0.8	0.6207
*p-value (follow-up* vs. *baseline)*	0.5952	0.3540	-
*Percentage changes from baseline*	3 ± 7	5 ± 8	0.3228
**Total prolactin** (ng/mL)			
*Baseline*	53.2 ± 10.2	51.8 ± 9.5	0.5973
*Follow-up*	50.6 ± 9.2	44.8 ± 10.3	**0.0305**
*p-value (follow-up* vs. *baseline)*	0.3210	**0.0107**	-
*Percentage changes from baseline*	−5 ± 10	−13 ± 8	**0.0017**
**Monomeric prolactin** (ng/mL)			
*Baseline*	49.2 ± 9.8	48.1 ± 8.2	0.6506
*Follow-up*	46.8 ± 8.9	41.6 ± 10.0	**0.0447**
*p-value (follow-up* vs. *baseline)*	0.3417	**0.0103**	-
*Percentage changes from baseline*	−5 ± 10	−14 ± 7	**0.0003**
**Macroprolactin** (ng/mL)			
*Baseline*	4.0 ± 2.8	3.7 ± 2.2	0.6575
*Follow-up*	3.8 ± 2.6	3.2 ± 1.8	0.3198
*p-value (follow-up* vs. *baseline)*	0.7829	0.3561	-
*Percentage changes from baseline*	−5 ± 10	−13 ± 25	0.1218
**FSH** (U/L)			
*Baseline*	3.5 ± 1.0	3.7 ± 1.2	0.5010
*Follow-up*	3.8 ± 1.2	4.3 ± 1.2	0.1248
*p-value (follow-up* vs. *baseline)*	0.3144	0.0668	-
*Percentage changes from baseline*	9 ± 12	16 ± 14	**0.0422**
**LH** (U/L)			
*Baseline*	3.0 ± 1.4	2.9 ± 1.2	0.7802
*Follow-up*	3.2 ± 1.0	3.6 ± 1.5	0.2455
*p-value (follow-up* vs. *baseline)*	0.5411	0.0591	-
*Percentage changes from baseline*	7 ± 11	24 ± 18	**0.0001**
**ACTH** (pg/mL)			
*Baseline*	35.2 ± 14.0	40.0 ± 12.5	0.1816
*Follow-up*	38.3 ± 16.0	41.1 ± 14.3	0.4928
*p-value (follow-up* vs. *baseline)*	0.4437	0.7604	-
*Percentage changes from baseline*	9 ± 20	3 ± 10	0.1614
**IGF-1** (ng/mL)			
*Baseline*	201 ± 55	189 ± 58	0.4304
*Follow-up*	195 ± 48	200 ± 60	0.7317
*p-value (follow-up* vs. *baseline)*	0.6654	0.4885	-
*Percentage changes from baseline*	−3 ± 18	6 ± 26	0.1379
**hsCRP** (mg/L)			
*Baseline*	3.4 ± 1.2	2.7 ± 1.0	**0.0213**
*Follow-up*	2.6 ± 0.7	1.2 ± 0.5	**<0.0001**
*p-value (follow-up* vs. *baseline)*	**0.0036**	**<0.0001**	**-**
*Percentage changes from baseline*	−24 ± 14	−56 ± 23	**<0.0001**

Group 1: hyperprolactinemic women with autoimmune thyroiditis; Group 2: hyperprolactinemic women without thyroid pathology. The data are presented as the mean ± standard deviation. Percentage changes from baseline were calculated by dividing the change from baseline by the absolute value of the baseline value and multiplying the result by 100. Statistically significant results are marked in bold. Reference values for young women in the early follicular phase: glucose: 70–99 mg/dL; HOMA1-IR: <2.0; TPOAb: <35 U/mL; TgAb: <35 U/mL; thyrotropin: 0.4–4.5 mU/L; free thyroxine: 10.2–21.3 pmol/L; free triiodothyronine: 2.2–6.4 pmol/L; total prolactin: 5.0–29.0 ng/mL monomeric prolactin: 4.0–26.0 ng/mL; macroprolactin: 2.0–4.0 ng/mL; FSH: 3.1–10.0 U/L; LH: 2.2–8.5 U/L; ACTH: 15–70 pg/mL; IGF-1: 85–300 ng/mL; hsCRP: <1.0 mg/L. Abbreviations: ACTH—adrenocorticotropic hormone; FSH—follicle-stimulating hormone; HOMA1-IR—the homeostatic model assessment 1 of insulin resistance ratio; hsCRP—high-sensitivity C-reactive protein; IGF-1—insulin-like growth factor-1; LH—luteinizing hormone; TgAb—thyroglobulin antibodies; TPOAb—thyroid peroxidase antibodies.

## Data Availability

The data that support the findings of this study are available from the corresponding author upon reasonable request.

## Abbreviations

ACTH—adrenocorticotropic hormone; FSH—follicle-stimulating hormone; HOMA1-IR—the homeostatic model assessment 1 of insulin resistance ratio; hsCRP—high-sensitivity C-reactive protein; IGF-1—insulin-like growth factor-1; LH—luteinizing hormone; TgAb—thyroglobulin antibodies; TPOAb—thyroid peroxidase antibodies.
